# An Address to the Members of the Missouri Dental Association

**Published:** 1866-09

**Authors:** H. E. Peebles

**Affiliations:** President


### AN ADDRESS TO THE MEMBERS OF THE MISSOURI DENTAL ASSOCIATION.

BY DR. H. E. PEEBLES, PRESIDENT.

We have perused this communication of Dr. P. with much interest, and are much pleased at the professional progress indicated by it in Missouri. The almost total want of effort by the profession in Missouri, which the Doctor seems so much to lament, hardly finds a verification in the opinions which we have entertained upon the subject. We have always felt that the profession, at least in St. Louis and vicinity, were abreast with the foremost in progress and development, and this, we think, is a very general opinion.

The organization of a State Association is a move in the right direction (every State *must* have its society), and will result in great good, not only in the State, but will serve as a stimulus upon the whole profession.

We learn from this that an effort is also being made to establish a Dental College in St. Louis. We presume, however, that it will be in connection with one of the medical schools.

More dental colleges are needed to answer the real requirements of the profession. But to the union of a dental with a medical school there has been much objection. Much care, doubtless, should be exercised in organizing a dental college under such an arrangement, but under favorable circumstances it may be done. We shall be glad to record the St. Louis Dental College as a *fixed fact.* This address will doubtless stir up the profession in Missouri to the performance of their duty. It is also gratifying to note that a movement is on foot to secure protection by legal enactment, and we doubt not the effort will be attended with success. Let progress be the watchword.

